# Diversity Arrays Technology (DArT) for Pan-Genomic Evolutionary Studies of Non-Model Organisms

**DOI:** 10.1371/journal.pone.0001682

**Published:** 2008-02-27

**Authors:** Karen E. James, Harald Schneider, Stephen W. Ansell, Margaret Evers, Lavinia Robba, Grzegorz Uszynski, Niklas Pedersen, Angela E. Newton, Stephen J. Russell, Johannes C. Vogel, Andrzej Kilian

**Affiliations:** 1 Department of Botany, The Natural History Museum, London, United Kingdom; 2 Diversity Arrays Technology P/L, Canberra, Australia; University of Texas Arlington, United States of America

## Abstract

**Background:**

High-throughput tools for pan-genomic study, especially the DNA microarray platform, have sparked a remarkable increase in data production and enabled a shift in the scale at which biological investigation is possible. The use of microarrays to examine evolutionary relationships and processes, however, is predominantly restricted to model or near-model organisms.

**Methodology/Principal Findings:**

This study explores the utility of Diversity Arrays Technology (DArT) in evolutionary studies of non-model organisms. DArT is a hybridization-based genotyping method that uses microarray technology to identify and type DNA polymorphism. Theoretically applicable to any organism (even one for which no prior genetic data are available), DArT has not yet been explored in exclusively wild sample sets, nor extensively examined in a phylogenetic framework. DArT recovered 1349 markers of largely low copy-number loci in two lineages of seed-free land plants: the diploid fern *Asplenium viride* and the haploid moss *Garovaglia elegans*. Direct sequencing of 148 of these DArT markers identified 30 putative loci including four routinely sequenced for evolutionary studies in plants. Phylogenetic analyses of DArT genotypes reveal phylogeographic and substrate specificity patterns in *A. viride*, a lack of phylogeographic pattern in Australian *G. elegans*, and additive variation in hybrid or mixed samples.

**Conclusions/Significance:**

These results enable methodological recommendations including procedures for detecting and analysing DArT markers tailored specifically to evolutionary investigations and practical factors informing the decision to use DArT, and raise evolutionary hypotheses concerning substrate specificity and biogeographic patterns. Thus DArT is a demonstrably valuable addition to the set of existing molecular approaches used to infer biological phenomena such as adaptive radiations, population dynamics, hybridization, introgression, ecological differentiation and phylogeography.

## Introduction

The development of innovative methods for detecting genetic variation has progressively enhanced the study of evolutionary relationships and processes [1]. High-throughput genomic tools such as the DNA microarray platform have sparked a remarkable increase in data production, leading to new evolutionary insights [2]. Their application is however nearly exclusively restricted to model and near-model organisms [2], [3]. In contrast, the detection of genetic variation in non-model plants and animal—the majority of life on earth—is largely restricted to direct sequencing of previously-identified variable loci or arbitrarily amplified dominant (AAD) markers, especially RAPDs, ISSRs and AFLPs [4].

Diversity Arrays Technology (DArT) was developed to overcome these and other limitations that prevent the application of microarray technology to non-model organisms. DArT is a hybridization-based genotyping technology which is currently implemented on the microarray platform to rapidly and simultaneously identify and type DNA polymorphism [5]. DArT detects primarily dominant markers, mostly resulting from single nucleotide polymorphisms at restriction sites [6] at hundreds to thousands of arbitrary genomic loci [6], [7]. First used to infer the genetic diversity in cultivated varieties of rice (*Oryza sativa* L.) [8], DArT was subsequently applied to barley (*Hordeum vulgare* L.) [7], grand eucalyptus (*Eucalyptus grandis* Hill ex Maiden) [9], cassava (*Manihot esculenta* Crantz) [10], wheat (*Triticum aestivum* L.) [11] and validated in the model organism thale cress (*Arabidopsis thaliana* L. Heynh) [6].

The application of DArT has so far been restricted to detection and mapping of DNA polymorphism in cultivated varieties of agriculturally important angiosperms. While some wild crop relatives have been included in previous DArT studies [10], [12], [13], the potential of DArT has not yet been explored in exclusively wild sample sets, nor extensively examined in a phylogenetic framework. Furthermore, because all existing DArT studies have been applied to flowering plants, its applicability to the rest of the plant kingdom and other organisms requires validation.

To explore whether DArT data can contribute to characterising evolutionary patterns and processes, we selected two lineages of seed-free land plants that provide two distinct opportunities to infer evolutionary hypotheses and detect hybridization by employing DArT: 1) the evolution of substrate specificity in the diploid fern *Asplenium viride* L. and 2) the phylogeography of the haploid moss *Garovaglia elegans* Dozy & Molk. Hampe ex Bosch & Sande Lac. These organisms were selected based on accessibility to well-preserved plant material and prior knowledge obtained by studying these organisms using conventional molecular evidence (allozymes and/or DNA sequencing). Furthermore, evolutionary interpretations have been hampered in both organisms by low DNA sequence diversity of a small number of selected loci.

## Materials and Methods

### Preparation and evaluation of DArT Discovery Libraries

Independent DArT discovery arrays were constructed for two 16-specimen study groups: (1) 14 specimens of the diploid fern *Asplenium viride*, one specimen of diploid *A. trichomanes* L. to polarize genetic structuring within *A. viride* and one specimen of their naturally derived allotetraploid hybrid, *A. adulterinum* J. Milde [14], and (2) 15 specimens of the haploid moss *Garovaglia elegans,* collected from various locations in Australia and New Guinea, and one specimen of *G. powellii* Mitt. for polarization (Tables 1 and 2). At least 1 µg of total genomic DNA was extracted from silica gel desiccated (*Asplenium*) or air dried (*Garovaglia*) leaf material using a modification of the standard CTAB procedure [15] as specified in Trewick *et al.* [16] except that extractions were incubated in 500 µL CTAB buffer, 50 µl sarkosyl and 10 µl proteinase-K and purified by phenol-chloroform extraction. DNA quantity, quality and competence for restriction endonuclease digestion were confirmed by visualizing 2 µL of total genomic DNA alongside 2 µL of *Mse*I restriction digested total genomic DNA by agarose gel electrophoresis and ethidium bromide staining. Genomic representations were produced from a mixture of either *Asplenium* or *Garovaglia* DNA samples according to Wenzl *et al.* [7]. Libraries were prepared as described [7], [8] either directly from PCR amplification products or from normalized amplification products subtracted using a modified version of suppression subtractive hybridization (SSH) [17] as described below. Polymorphic markers detected on the *Asplenium* and *Garovaglia* discovery arrays were re-arrayed onto “genotyping arrays” to enable genotyping of the individual samples. The *in vivo* genomic copy number of each DArT marker was approximated by hybridizing un-amplified metagenomic DNA to each genotyping array.

**Table 1:** *Asplenium* samples used in this study with voucher information, collecting locality, substrate specificity, ploidy level and GenBank accession numbers.

sample	voucher info.	locality	substrate	ploidy	*trn*L-F	*rps4-trn*S	*pgi*C
***Asplenium viride*** ** L.**							
Avi69	69 (BM^a^)	France	limestone	di-	EF645609	EF645625	EF645641
Avi70	70C (BM^a^)	Norway	limestone	di-	EF645602	EF645618	EF645634
Avi169	not vouchered	Norway	serpentine	di-	EF645604	EF645620	EF645636
Avi245	245(6) (BM^a^)	Morocco	limestone	di-	EF645608	EF645624	EF645640
Avi255	255C (BM^a^)	Canada	limestone	di-	EF645598	EF645614	EF645630
Avi268	268 (BM^a^)	Austria	limestone	di-	EF645600	EF645616	EF645632
Avi272	272C (BM^a^)	Austria	serpentine	di-	EF645603	EF645619	EF645635
Avi281	281 (BM^a^)	Croatia	limestone	di-	EF645601	EF645617	EF645633
Avi284a	284(1) (BM^a^)	Austria	magnesit	di-	EF645606	EF645622	EF645638
Avi284b	284A(2) (BM^a^)	Austria	magnesit	di-	EF645607	EF645623	EF645639
Avi288^b^	288 (BM^a^)	Switzerland	serp.-lime. cglm.	di-	EF645610	EF645626	EF645642
Avi289^b^	289 (BM^a^)	Switzerland	serp.-lime. cglm.	di-	EF645611	EF645627	EF645643
Avi291	291A (BM^a^)	UK	serpentine	di-	EF645605	EF645621	EF645637
Avi293	293A (BM^a^)	Canada	limestone	di-	EF645599	EF645615	EF645631
***Asplenium trichomanes*** ** L.**							
Atr120	120 (BM^a^)	Canada	unknown	di-	EF645613	EF645629	EF645644
***Asplenium adulterinum*** ** J. Milde**							
Aad79	79B (BM^a^)	Austria	serpentine	tetra-	EF645612	EF645628	N/A^c^

a Unmounted specimens in the Molecular Lab Herbarium.

b Avi288 and Avi289 were included in the *Asplenium* metagenome for the discovery, typing and quantitative evaluation of DArT markers, but were excluded from subsequent analyses due to substrate ambiguity.

c As expected for an allopolyploid hybrid, the nuclear locus *pgiC* exhibits heterozygosity at the DNA sequence level and was thus not submitted to GenBank.

**Table 2:** *Garovaglia* samples used in this study with voucher information, collecting locality, substrate specificity, ploidy level and GenBank accession numbers.

sample	voucher info	locality	*trn*G
***Garovaglia elegans*** ** (Dozy & Molk.) Bosch & Sande Lac. subsp. ** ***dietrichiae*** ** (Müll. Hal.) During**			
Gel5465	Newton 5465 (BM^1^)	Australia	DQ194243
Gel6446	Newton 6446 (BM^1^)	Australia	EF551190
Gel6434^a^	Newton 6434 (BM^1^)	Australia	not sequenced
Gel6485	Newton 6485 (BM^1^)	Australia	not sequenced
Gel6504	Newton 6504 (BM^1^)	Australia	EF551192
Gel6516	Newton 6516 (BM^1^)	Australia	EF551196
Gel6520	Newton 6520 (BM^1^)	Australia	EF551193
Gel6524	Newton 6524 (BM^1^)	Australia	EF551198
Gel6532	Newton 6532 (BM^1^)	Australia	EF551191
Gel6547	Newton 6547 (BM^1^)	Australia	EF551197
Gel6550	Newton 6550 (BM^1^)	Australia	EF551194
Gel6560	Newton 6560 (BM^1^)	Australia	EF551195
***Garovaglia elegans*** ** subsp. ** ***elegans*** ** (Müll. Hal.) During**			
Gel42.588	Sloover 42.588 (NY)	Papua New Guinea	EF551189
***Garovaglia elegans*** ** fo. ** ***latifolia*** ** (E.B. Bartram) During**			
Gel40397	Streimann 40397 (NY)	Papua New Guinea	EF551188
Gel40482	Streimann 40482 (NY)	Papua New Guinea	EF551187
***Garovaglia powellii*** ** ssp. ** ***muelleri*** ** (Hampe) During**			
Gpo6496	Newton 6496 (BM^1^)	Australia	DQ194245

a Gel6434 was discovered to be derived from a mixed-taxon cushion containing individuals of both *G. elegans* ssp. *dietrichiae* and *G. powelli.*

### Genotyping and analysis of DArT images

The genotypes of all individual *Asplenium* and *Garovaglia* samples were scored as absence/presence for 444 and 905 markers, respectively, and formatted as a binary data matrix (Table S1). The reproducibility of the two DArT genotyping arrays was examined by independent assay using the same DNA. The genotyping of genomic representations of individual samples was performed substantially as described [7], with the exception that the polylinker fragment (reference in DArT assay) was labelled with FAM instead of Cy5 dye. Arrays were scanned with 10 µm resolution at 543 nm (Cy3) and 488 nM (FAM) on a LS300 confocal laser scanner (Tecan, Grödig, Austria) as described in Akbari *et al.* [11]. Array images were analyzed with DArTsoft 7.4 (Diversity Arrays Technology P/L, Canberra, Australia). The program automatically recognized array features using a seeded-region-growth algorithm and reported, for each fluorescent channel, the average and standard deviation (SD) of pixel intensities within and around each array feature, the fraction of saturated pixels within each feature and the number of pixels of each feature, amongst other parameters (C. Cayla, personal communication). Clones with variable relative hybridization intensity across slides were subjected to fuzzy k-means clustering to convert relative hybridization intensities into binary scores (presence vs. absence). The quality of each marker was then determined using several parameters including 1) p-value, the variance of the relative target hybridization intensity between allelic states as a percentage of the total variance, 2) call rate, the percentage of DNA samples with binary (1 or 0) allele calls and 3) reproducibility, the fraction of concordant calls for replicate assays (C. Cayla, personal communication). Samples with cluster membership probability calculated by DArTsoft's clustering algorithm below the threshold of 0.8 were not classified (“X”). The frequencies of *Asplenium* and *Garovaglia* DArT markers with at least one X score across the sample set were 178 and 1 respectively. This discrepancy may be explained by differences in ploidy between *Asplenium* (diploid sporophyte) and *Garovaglia* (haploid gametophyte) because scoring efficiency would be expected to diminish as a result of intermediate signal intensities corresponding to copy number variation in polyploid samples. Indeed, the majority (56%) of X scores in the *Asplenium* data set were detected in the genotype of the known allotetraploid F1 hybrid sample of *A. adulterinum*.

### Suppression subtractive hybridization (SSH)

SSH was performed using a modification of the protocol given by Diatchenko *et al.* [17]. *Asplenium viride* sample Avi284b and *G. elegans* ssp. *dietrichiae* sample Gel6504 were selected arbitrarily as subtraction drivers for the *Asplenium* and *Garovaglia* experiments respectively. The following different mixtures (“testers”) of *Asplenium* and *Garovaglia* samples were used: *Asplenium* tester 1 (all *A. viride* amplification products excluding the driver Avi284b), *Asplenium* tester 2 (all *Asplenium* amplification products excluding the driver Avi284b) and *Garovaglia* tester (all *Garovaglia* amplification products excluding the driver Gel6504). All *Asplenium* and *Garovaglia* tester and driver digestion products were phenol chloroform extracted, isopropanol precipitated and resuspended in deionized water. Digested testers were ligated to adapters with either Core 1 or Core 2, corresponding to the relevant digesting enzymes, in separate reactions (Table 3). Subtraction was carried out in either one or two stages. 600 ng driver was mixed with 20 ng tester-Core 1 and 20 ng tester-Core 2, isopropanol precipitated and resuspended in Subtraction Hybridization Buffer (50 mM HEPES pH 8.3, 0.5 M NaCl, 0.02 M EDTA pH 8.0, 10% PEG 8000 w/v). The dissolved pellet was denatured and hybridized for 5 hr at 68°C (for two-stage subtraction, 300ng freshly denatured driver was added and hybridized for a further 5 hr at 68°C). Subtraction products were used as a template in an amplification reaction using Core 1 and 2 primers (Core 1 5′-GAGTAGTGCCAGAACGGTC-3′, Core 2 5′-TCGTAGACTGCGTATCCG-3′).

**Table 3:** Subtraction adapters (5′ to 3′).

DpnII	Core 1	GATCGACCGTTCTGGCA annealed to CTGAGTAGTGCCAGAACGGTC
	Core 2	GATCCGGATACGCAGTCTA annealed to CCTCGTAGACTGCGTATCCG
HpyCH4IV	Core 1	GCGACCGTTCTGGCA annealed to CTGAGTAGTGCCAGAACGGTC
	Core 2	CGCGGATACGCAGTCTA annealed to CCTCGTAGACTGCGTATCCG
MseI	Core 1	TAGACCGTTCTGGCA annealed to CTGAGTAGTGCCAGAACGGTC
	Core 2	TACGGATACGCAGTCTA annealed to CCTCGTAGACTGCGTATCCG
NlaIII	Core 1	CTGAGTAGTGCCAGAACGGTCCATG annealed to GACCGTTCTGGCA
	Core 2	CCTCGTAGACTGCGTATCCGCATG annealed to CGGATACGCAGTCTA

### Quantitative evaluation of DArT data

Polymorphism information content (PIC) [18], P and Q, reproducibility and call rate (C. Cayla, personal communication) were examined for both *Asplenium* and *Garovaglia* datasets to assess the distribution and reliability of hybridization (Table S1). The distribution of *Asplenium* DArT polymorphisms was examined on a per species basis according the following four categories: *A. trichomanes* private, *A. viride* private, *A. trichomanes*/*A. viride* shared, or *A. viride* “X”s but no “1”s. The *A. adulterinum* genotype was excluded from this process, as it contained polymorphisms shared by and private to both *A. viride* and *A. trichomanes*. Also excluded were 84 subtraction-derived DArT markers in the *Asplenium* data set that displayed an identical scoring pattern across all samples (this pattern did not match any known geographic patterns or systematic relationships and, when these 84 markers were included in a comparison of the diversity patterns between subtracted and non-subtracted genomic representations, substantial differences in inferred relationships were observed). Samples Avi288 and Avi289 were also excluded due to substrate ambiguity. The distribution of *Garovaglia* DArT polymorphisms was similarly examined on a per species basis according the following four categories: *G. powellii* private, *G. elegans* ssp. *dietrichiae* (Australia) private, *G. elegans* ssp. *elegans* and *G. elegans* fo. *latifolia* (Papua New Guinea) shared, or unassignable by taxonomy or geography. The *G. elegans* sample Gel6434 was excluded from this process, as it was derived from a mixed-taxon cushion, containing individuals of both *G. elegans* ssp. *dietrichiae* and *G. powellii*.

### Direct sequencing of chloroplast and nuclear loci

For *Asplenium*, total genomic DNA was extracted from silica desiccated leaf material as described above. The following portions of the chloroplast genome were amplified by the polymerase chain reaction: the combined *trn*L(_CAA_) gene and *trn*L*-trn*F(_GAA_) intergenic spacer (IGS) region (*trn*L-F), using primers FERN1 [16] and F [19] as specified in Trewick *et al.* [16] and the combined *rps4* gene and *rps4-trn*S(_GAA_) IGS region (*rps4-trn*S) following the primers and conditions specified in Schneider *et al.* [20]. Exons 14 to 16 of the single-copy nuclear locus *pgi*C were amplified using primers 14F and16R according to Ishikawa *et al.* [21]. Bidirectional cycle sequencing was carried out on an ABI 3730 capillary DNA sequencer using ABI PRISM BigDye Terminator Cycle Sequencing Ready Reaction Kit (Perkin-Elmer) and the same primers used for amplification. Sequence contigs were assembled and automatically aligned with subsequent manual corrections using SeqMan and MegAlign respectively (v. 6.00 Lasergene, DNAstar, Madison, WI, USA). For *Garovaglia*, total genomic DNA was extracted using a modification of the standard CTAB procedure [15] as specified in Pedersen and Newton [22]. The chloroplast encoded *trn*G(_UCC_) intron (*trn*G) was amplified, sequenced and aligned using primers and conditions as specified in Pedersen and Newton [22]. All sequences were deposited in GenBank (Tables 1 and 2).

### Direct sequencing of selected DArT markers

Inserts and part of the polylinker region were amplified for a representative selection of 96 DArT marker clones from each study using M13 forward and reverse primers as reported in Jaccoud *et al* [8]. Amplification products were bidirectionally sequenced as described above using the same primers used for amplification. Successful sequence reads were obtained for 74 *Asplenium* and 74 *Garovaglia* DArT markers. Sequence contigs were assembled (vectors removed) and aligned as described above. All sequences were deposited in GenBank (Table S1).

### Sequence similarity searches and redundancy estimates

Each of the 148 DArT marker sequences were queried against 1) all other sequenced DArT markers in the same study and 2) GenBank (www.ncbi.nlm.nih.gov) to identify putative sequence identities using the Basic Local Alignment Search Tool [23] for nucleotides (blastn) and translated to proteins (tblastx). For inclusion in Tables S1 and 4 we applied an arbitrary maximum expect value of 10^−4^ and an arbitrary minimum of 70% identity at the nucleotide sequence level or two or more alignments to the same locus/function in different plant species.

### Phylogenetic analyses of DArT data and DNA sequence data

Because the standard DArT protocol (DNA not sheared, not subtracted) yielded the most consistent data across plates and also produced the greatest number of DArT markers of any of the treatments, only DArT markers produced using the standard treatment were used in phylogenetic analyses. *Asplenium* samples Avi288 and Avi289 were excluded due to substrate ambiguity (Table 1). To infer relationships, a binary matrix was generated in which absence (0) or presence (1) of DNA fragments was scored (Table S1). Both DArT data sets (*Asplenium* and *Garovaglia*) were analysed using principal component analyses (PCA) and principal coordinate analyses (PCoA) with Euclidean distances using the software packages PAST [24] and MVSP (Kovach Computing, Pentraeth, UK). In the case of *Garovaglia* data, a hierarchical PCoA procedure was performed in which the most distant specimens were excluded step-by-step to obtain a higher resolution in the scatter plots for the more closely related specimens. Phylogeny reconstructions were carried out using either distance-based approaches with LogDet and Nei-Li distance corrections or character based approaches, for example Bayesian inference of phylogeny with the MK1 model, maximum parsimony with equally weighted and unordered characters. NeighborNet analyses were employed to explore putative alternative relationships. Evolution of substrate specificity was reconstructed using the consensus topology based on all trees sampled from the plateau phase of the Bayesian analyses carried out with MrBayes. Both maximum parsimony and maximum likelihood approaches were applied to reconstruct changes in substrate preferences. These analyses were carried out with the appropriate software MacClade [25], Mesquite [25], MrBayes [26], PAUP* 4.0 [27], Splitstree [28], Tracer [29], and Treecon [30]. DNA sequence data were assembled and aligned using the Lasergene software package (DNASTAR, Madison, WI, USA) and MacClade. Phylogenetic hypotheses based on sequence data were generated using PAUP and MrBayes.

## Results

The frequencies of polymorphic markers detected on the *Asplenium* and *Garovaglia* discovery arrays were 6% and 15% respectively, resulting in 444 and 905 polymorphic markers recovered respectively (Table 5). The reproducibility of the two DArT genotyping arrays was successfully validated by independent assays from the same DNA (unpublished data). The polymorphism information content [18] for each marker is shown in Table S1; with average PIC (0.21 for *Asplenium*, 0.25 for *Garovaglia*), lower than expected for randomly chosen bi-allelic loci (0.50) and lower than in previous DArT studies in barley (0.38) [7] and cassava (0.42) [10].

To examine taxonomic specificity of DArT markers and to evaluate DArT protocol variables, especially enrichment by suppression subtractive hybridization (SSH), *Asplenium* and *Garovaglia* DArT markers (Table S1) were classified according to their distribution among species. *Asplenium* DArT markers were distributed as follows: *A. trichomanes* private (65%), *A. viride* private (16%), *A. trichomanes*/*A. viride* shared (13%) and *A. trichomanes/A. viride* putatively shared (present in *A. trichomanes*, “X” in *A. viride*, 5%). *Garovaglia* DArT marker presence was distributed as follows: *G. powellii* private (30%), *G. elegans* ssp. *dietrichiae* private (33%), *G. elegans* ssp. *elegans* and/or *G. elegans* fo. *latifolia* private (19%) unassignable by taxonomy (18%).

The fraction of *Asplenium* DArT markers in each of these categories varied according to treatment: DArT markers derived from the pooled *Asplenium* metagenome using the standard DArT protocol (no shearing, no SSH) (*Asplenium* plates 1–6, n = 103) were distributed as follows: *A. trichomanes* private (58%), *A. viride* private (12%), *A. trichomanes*/*A. viride* shared (20%) and *A. trichomanes/A. viride* putatively shared (10%). By contrast, DArT markers derived solely from *A. viride* genomic DNA driver Avi284b (*Asplenium* plates 7–8, n = 22) were enriched for *A. viride* private (50%) and *A. trichomanes*/*A. viride* shared (50%) polymorphisms. In the reverse treatment, *A. viride* Avi284b genomic DNA was subtracted from the pooled *Asplenium* metagenome (*Asplenium* plates 11–12 & 15–16, n = 193) and the resulting dataset was enriched for *A. trichomanes* private polymorphisms (86%). Lastly, when *A. viride* Avi284b genomic DNA was subtracted from a sub-metagenome containing only *A. viride* genomic DNA samples except Avi284b (*Asplenium* plates 9–10 & 13–14, n = 35), the proportion of *A. trichomanes* private polymorphisms was reduced (9%) and *A. viride* private polymorphisms increased (71%).

The fraction of *Garovaglia* DArT markers in each category also varied according to treatment: DArT markers derived from the pooled *Garovaglia* metagenome using the standard DArT protocol (no shearing, no SSH, *Garovaglia* plates 1–8, n = 262) were distributed as follows: *G. powellii* private (15%), *G. elegans* ssp. *dietrichiae* private (32%), *G. elegans* ssp. *elegans* and/or *G. elegans* fo. *latifolia* private (31%) or unassignable by taxonomy or geography (23%). By contrast, DArT markers derived solely from *G. elegans* genomic DNA driver Gel6504 (*Garovaglia* plates 15–16, n = 55) were enriched for *G. elegans* ssp. *dietrichiae* private (35%) and *G. elegans* ssp. *elegans* and/or *G. elegans* fo. *latifolia* private (42%) polymorphisms. In the reverse treatment, *G. elegans* Gel6504 genomic DNA was subtracted from the pooled *Garovaglia* metagenome (*Garovaglia* plates 9–14, n = 413) and the resulting dataset was enriched for *G. powellii* private polymorphisms (44%).

To detect DArT marker specificity by substrate or geography, we examined the distribution of DArT markers in substrate-specific or geographically defined *Asplenium* and *Garovaglia* specimens. In the complete unsubtracted *Asplenium* data set (*Asplenium* plates 1–6), the *A. viride* private marker class included 1 global limestone-specific DArT marker (marker ID *Asplenium*6F4), 4 serpentine-specific DArT markers, and 7 non-substrate-specific DArT markers; the *A. trichomanes*/*A. viride* shared marker class included 6 DArT markers absent from global limestone and 1 DArT marker absent from serpentine (marker ID *Asplenium*3O24). In the complete unsubtracted *Garovaglia* data set (*Garovaglia* plates 1–8, Table S1), 33% of DArT markers are specific to Australian *G. elegans* samples while 19% are specific to Papua New Guinean *G. elegans* samples.

The genotype of *G. elegans* sample Gel6434 contains both *G. powellii*- and *G. elegans* (Australia)-private DArT markers, potentially consistent with a hybrid origin. Perplexingly, this accession was not a known or suspected hybrid. Re-examination of the cushion from which this accession was dissected revealed that it was a mixed-taxon cushion containing individuals of both *G. elegans* ssp. *dietrichiae* and *G. powellii*. Gel6434 was therefore retroactively excluded from the marker distribution analyses of all of the other markers reported above.

The copy number and genomic origin of any marker system, and the potential for redundancy between markers, is an important consideration for evolutionary applications. Only approximately 15% of *Asplenium* DArT markers hybridized to their respective un-amplified, labelled metagenomes (unpublished data) indicating that approximately 85% of *Asplenium* DArT markers are low-copy sequences. The level of redundancy between supposedly independent DArT markers on each genotyping array, as well as between SSH treatments, was assessed by sequencing a selection of 74 *Asplenium* and 74 *Garovaglia* DArT markers, (Table S1, summarized in Table 4). This revealed that 10.7% of *Asplenium* DArT markers and 16.2% of *Garovaglia* DArT markers shared over 99% identity at the nucleotide sequence level to other DArT markers (Table 5). Redundancy dropped to 3.3% and 5.6% respectively when SSH-derived DArT markers were removed. Similar redundancy estimates, including lower redundancy in un-subtracted libraries, are reported for wheat [11], tomato, sorghum and sugarcane (DArT P/L, unpublished).

**Table 4:** Predicted locus/function for sequenced DArT markers with >70% identity to one or more GenBank sequences or (^a^and) >1 plant species hit for the same function.

DArT marker	length	predicted function or locus
*Asplenium*		
2 B8	442bp	expressed gene (similarity to cDNA, mRNA and/or protein)^a^
2 G20	581	expressed gene (possible retrotransposon)
2 H15	691	cyclophilin-like protein (CYP20)^a^ (redundant with *Asplenium* 8 E23)
2 K16	708	*Hcr2*
2 M10	852	expressed gene (similarity to cDNA, mRNA and/or protein)^a^
3 I22	704	expressed gene (similarity to cDNA, mRNA and/or protein)
4 A18	537	GGPP synthase
5 H4	460	methyltransferase^a^
7 E12	627	eukaryotic translation initiation factor 3B (EIF3B)^a^
7 M10	640	*cyc07* and/or *rps3* ^a^
7 O5	527	expressed gene (similarity to cDNA, mRNA and/or protein)
8 E23	698	cyclophilin-like protein (CYP20)^a^ (redundant with *Asplenium* 2 H15)
8 H10	340	glutamine synthetase^a^
12 D11	101	Chloroplast-encoded *trn*K-*psb*A-*trn*H^a^
12 G13	146	glyceraldehyde-3-phosphate dehydrogenase^a^
12 H16	124	Mitochondrial pseudogene *rpl2*
13 F23	173	carbohydrate transporter
16 C20	123	expressed gene (similarity to cDNA, mRNA and/or protein)^a^
*Garovaglia*		
1 D19	637	expressed gene, protein binding function^a^ (redundant with *Garovaglia* 5 C20)
1 E5	382	structural maintenance of chromosomes I (SMCI)^a^
1 K11	279	kinase
3 D6	333	expressed gene (similarity to cDNA, mRNA and/or protein)
4 M15	448	protein kinase^a^
4 P4	292	expressed gene (similarity to cDNA, mRNA and/or protein)
5 C20	635	expressed gene, protein binding function^a^ (redundant with *Garovaglia* 1 D19)
6 H21	598	expressed gene (similarity to cDNA, mRNA and/or protein)
7 H9	693	biotin synthase
9 K13	649	Chloroplast-encoded*16s* rRNA gene^a^
12 F16	155	polyribonucleotide nucleotidyltransferase (3'RNAse)^a^
13 B20	138	histidine kinase^a^
14 O1	118	expressed gene (similarity to cDNA, mRNA and/or protein)
14 O18	201	sugar transporter^a^

DArT markers are identified by discovery array plate number and location (row, column) in Table S1.

**Table 5:** Summary of quantitative evaluation of *Asplenium* and *Garovaglia* DArT markers (for individual markers see Table S1).

	*Asplenium*			*Garovaglia*		
	no SSH	SSH	total	no SSH	SSH	total
sheared	no	yes	-	no	yes	-
clones tested for polymorphism	3072	3072	6144	3840	2304	6144
polymorphic (DArT) markers recovered	126	318	444^a^	420	485	905
frequency of polymorphism	0.04	0.08	0.06	0.11	0.21	0.15
average reproducibility	97.85	97.63	97.71	98.73	99.3	99.03
average call rate	99.68	99.61	99.64	99.54	99.75	99.65
average PIC	0.24	0.19	0.21	0.27	0.23	0.25
DArT markers sequenced (attempted)	36 (49)	38 (47)	74 (96)	36 (49)	38 (47)	74 (96)
ave. length of sequenced DArT markers	539	176	344	480	182	327
DArT marker length range	292–852	82–565	82–852	279–789	37–649	37–789
frequency of redundancy	0.2	0.31	0.26	0.14	0.47	0.31

a For distribution and phylogenetic analyses, 84 of the 444 *Asplenium* DArT markers that displayed an identical scoring pattern across all samples analysed were excluded (see Materials and Methods).

BLAST searches of 74 *Asplenium* and 74 *Garovaglia* DArT marker sequences against GenBank recovered 18 (24%) and 14 (19%) significant alignments respectively, and included known mitochondrial (i.e. *rpl*2 mitochondrial pseudogene), chloroplast (i.e. *psb*A and *16s*) and nuclear (i.e. *Hcr2* and *cyc07/rps3*) loci as well as proteins (e.g. CYP20, EIF3B and G3PDH) and predicted expressed genes (Table 4).

**Figure 1:**
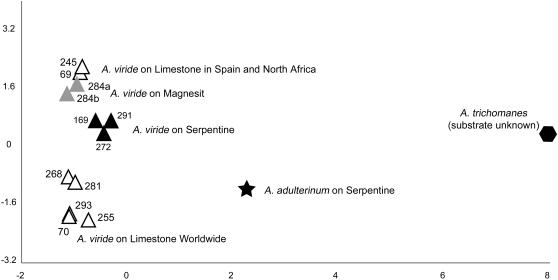
Two dimensional PCO scatter plot of the *Asplenium viride* DArT marker data set (triangles) plus a single specimen of *A. adulterinum* (star) and *A. trichomanes* (hexagon). Color of symbol corresponds to the substrate on which the sample was growing: limestone = white, serpentine = black and magnesit = gray. Numbers correspond to the sample number (Table 1).

**Figure 2:**
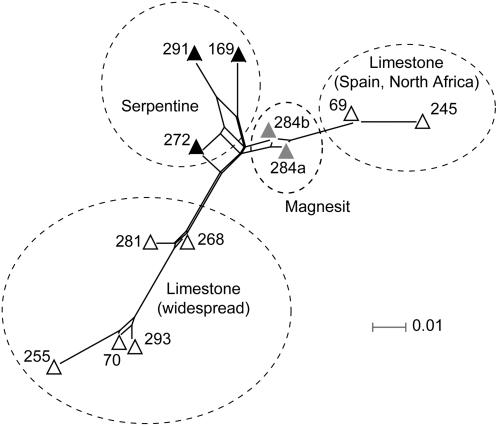
Splitgraph obtained by a NeighborNet analysis with LogDet distances for the *Asplenium viride* DArT marker set. Sample numbers, symbols and symbol colors as in Figure 1. The dotted ovals mark putative groups found in phylogenetic analyses (Figure 3).

PCO and PCoA analyses of the *Asplenium* and *Garovaglia* DArT data sets derived from the standard DArT procedure (not subtracted) recovered a cumulative explanatory percentage for axes 1 and 2 of 72.7% for *Asplenium* and 73.5% for *Garovaglia*, the latter increasing to 100% in the exclusively Papua New Guinean sample subset, but not in the exclusively Australian subset. These DArT data enabled reconstruction of intraspecific structure in *A. viride* revealing phylogeographic and substrate specificity patterns (Figures 1 & 2). These patterns were not detectable in our analyses of cpDNA or nrDNA sequences, due to insufficient polymorphisms to generate a fully resolved phylogeny (data not shown). The *rps*4-*trn*S IGS contained just two polymorphic positions, while the *trn*L-F region had one unambiguous polymorphic nucleotide position. In both cpDNA regions, one polymorphic site separates samples Avi169 and Avi272 from the remaining samples of *A. viride.* Variation in the nuclear *pgi*C data set was similarly low, with seven unambiguous polymorphic sites each of which was specific to only one specimen (e.g. Avi69, Avi245, Avi284a).

**Figure 3:**
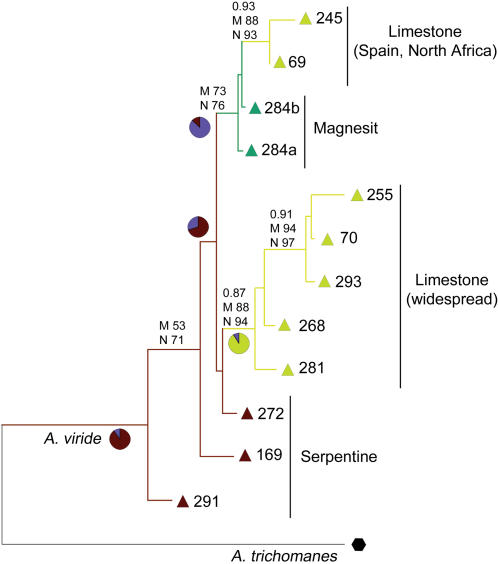
Consensus phylogram obtained using Bayesian inference of phylogeny analyses for the *A. viride* DArT marker data set rooted with *A. trichomanes*. Posterior probabilities (0.XX), maximum parsimony bootstrap values (M XX), and bootstrap values for a neighbor joining distance analyses with Nei-Li distances (N XX) are given above branches if they are >0.75 for posterior probabilities or >50% for bootstrap values. Sample numbers and symbols as in Figure 1. Color of symbol corresponds to the substrate on which the sample was growing: limestone = yellow, serpentine = dark red and magnesit = green. Branch color corresponds to the character state reconstructed using a maximum parsimony approach. The pie charts represent the likelihood of substrate preference for branches with a putative switch between limestone, magnesit, or serpentine substrates, shown as either limestone versus non-limestone or serpentine versus non-serpentine.

All analyses of the *Asplenium* DArT marker data indicate one group with a widespread limestone genotype and a second group containing a north-African/Iberian limestone genotype with the two magnesit samples from Austria as putative relatives (Figures 1, 2, 3). In PCO analyses, the three *A. viride* samples occurring on serpentine are closely associated, and form a putative group in some phylogenetic reconstructions, forming a basal grade at the base of the remaining *A. viride* groups in others. The three samples of serpentine *A. viride* were collected in three different regions of Europe: Scandinavia (Avi291), Scotland (Avi169) and Austria (Avi272). The *Asplenium* DArT marker data set suggests that serpentine was the ancestral substrate of *A. viride* with two independent colonizations onto limestone (Figure 3).

**Figure 4:**
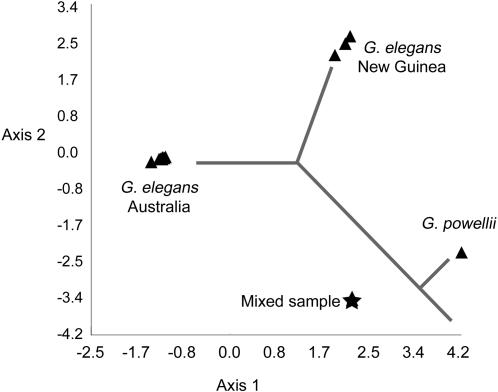
Two-dimensional PCO scatter plot of the *Garovaglia elegans* DArT marker data set superimposed with the phylogenetic tree obtained based on cpDNA. *Garovaglia powelli* is included to root the phylogenetic tree. Triangles correspond to single species samples, whereas the star corresponds to a mixed sample that includes individuals of *G. elegans* and *G. powellii*.

DNA sequence data from chloroplast (*Asplenium trn*L-F, *rps4*-*trn*S; *Garovaglia trn*G) and nuclear (*Asplenium pgi*C) loci largely corroborated the results of the above analyses of *Asplenium* and *Garovaglia* DArT data (Figures 2 & 4 and unpublished data). The analysis of *Garovaglia* chloroplast DNA sequence data resulted in a phylogram with a clear separation of Papua New Guinean and Australian specimens (Figure 4). The same clusters were recovered using *Garovaglia* DArT markers. Neither cpDNA nor the highly variable DArT markers recovered any phylogeographic patterns within Australia. In a hierarchical PCO analysis procedure (step by step exclusion of more distantly related samples), no increase in the cumulative percentage explained by axes 1 and 2 was observed.

In both *Asplenium* and *Garovaglia,* DArT markers enabled the detection of additive variation caused by hybridization (*A. adulterinum*) or a mixed-taxon specimen (*Garovaglia*). The genotype of *A. adulterinum*, a known allopolyploid *A. viride x trichomanes* hybrid, contains DArT markers that are private to both *A. trichomanes* and to the widespread limestone *A. viride* (Table S1). The position of *A. adulterinum* in the PCO analysis is intermediate between that of *A. trichomanes* and widespread limestone *A. viride* (Figure 1). Chloroplast DNA sequence data corroborate these DArT-based results: *A. adulterinum* exhibits an *A. viride* chloroplast haplotype (100% sequence identity with the common cp haplotype of *A. viride*), consistent with the known maternal inheritance of chloroplast DNA in *Asplenium* and that *A. viride* is always *A. adulterinum's* ovule parent [31], [32]. The genotype of the mixed-taxon *Garovaglia* sample Gel6434 contains DArT markers private to both *G. powellii* and the Australian *G. elegans* group.

## Discussion

This study demonstrates that the DArT protocol can be utilized to reproducibly detect largely low-copy genomic variation in sample sets from two lineages of seed-free land plants, suggesting that DArT may be useful across a broader taxonomic spectrum than that addressed in previous studies focusing on cultivated angiosperm species [6]–[11]. *Asplenium* and *Garovaglia* DArT data were useful for making biological inferences, for example, phylogeographic structure in plants with a high dispersal capacity, naturally occurring hybridization, exploration of chimeric environmental samples, and the reconstruction of ecological differentiation. Here we discuss procedures for both detecting and analysing DArT data for evolutionary applications, practical considerations for investigators, and the evolutionary hypotheses suggested by these results.

### Procedures for detecting DArT markers for evolutionary studies

The frequencies of polymorphism detected on the *Asplenium* (6%) and *Garovaglia* (15%) discovery arrays are similar to those reported for previous studies in cultivated angiosperms and their wild relatives: barley (2.9–10.4%) [12], cassava (9–14%) [10], mouse-ear cress (7.3%) [6]. The number of polymorphic DArT markers obtained from *Asplenium* (444) and *Garovaglia* (905) is a function of number DArT clones screened in the discovery array step, and the frequency of polymorphism therein. Because marker redundancy was estimated as less than 10% between DArT markers from un-subtracted metagenomic libraries, it is clear that several times as many DArT markers can be developed for the *Asplenium* or *Garovaglia* metagenomes through screening additional clones/arrays before saturation of marker libraries is reached.

That the average PIC values (0.21 for *Asplenium*, 0.25 for *Garovaglia*) are lower than previous DArT studies in barley (0.38) [7] and cassava (0.42) [10], and is likely due to the choice of sample sets with a strong structure, and clearly defined outgroups. All DArT markers private to the outgroups exhibited low PIC values as expected, and thus the high frequency of such markers impacted strongly on average PIC value.

Because the aim of this study was to pilot DArT in new taxonomic contexts and apply it to different classes of questions, a range of methodologies were compared to inform similar future studies. The distributions of DArT markers derived from sample-specific vs. pooled metagenomes exhibited expected shifts in marker distribution. For example, the DArT markers derived from one *A. viride* sample (Avi284b, *Asplenium* plates 7 & 8) or the *A. viride* sub-metagenome (*Asplenium* plates 9, 10, 13 & 14) were enriched for markers private to and shared with *A. viride* compared to the baseline marker set derived from the complete pooled *Asplenium* metagenome. Similarly, the distributions of DArT markers derived from subtracted vs. un-subtracted libraries exhibited expected shifts in marker distribution. For example, when Avi284b genomic DNA was subtracted from the *A. viride* sub-metagenome (all *A. viride* samples except Av284b), the number of *A. viride* private polymorphisms was enriched as expected. Thus restricting the starting metagenome and/or performing SSH allows investigators to intentionally enrich for DArT markers private to any one or more input samples in cases where such an enrichment is desired. However, when the maximum number of random markers is preferred, such as for unbiased evolutionary analyses, SSH is unnecessary for or even detrimental to the detection and typing of DArT markers in randomly derived sample sets.

### Procedures for analysing DArT markers to reconstruct the evolutionary history of plant species

The phylogenetic analyses of *Asplenium* and *Garovaglia* DArT data largely corroborated results generated using direct sequencing, and often improved upon the resolution provided by these methods. Considering the small sample set used in these pilot studies, our result holds much promise for the use of DArT to address evolutionary-genetic hypotheses when large sample sets are employed. The two case studies were designed to demonstrate the utility of DArT markers for studies in which highly variable markers are required for evolutionary interpretation and in which relationships are poorly resolved using DNA sequence data due to a low level of variation. Various other marker systems, isozymes and arbitrarily amplified dominant (AAD) markers for example, have been employed to overcome this problem [4], [33], [34]. DArT markers share several features with AAD markers but also differ substantially in other respects. DArT markers and AAD markers have the same binary data structure, that is, presence or absence of markers or bands and a dominant inheritance. Here we discuss some of the relevant distinguishing features of DArT data in comparison with AAD markers, and recommend how DArT data should be handled in phylogenetic or phylogeographic studies.

Non-independence of markers and false homology of bands are common problems associated with AAD markers. These drawbacks restrict the application of AAD markers to very closely related samples (e.g. within species) and often necessitate a dense sampling of the lineages to infer evolutionary history [4]. Because DArT markers are detected by DNA-DNA hybridization rather than fragment size, DArT markers were expected to suffer only a very low level of erroneous homology assessment/assignment. This assumption was validated by Wenzl *et al.* [12] through genetic mapping of over 2000 DArT markers in many mapping populations of barley, where fewer than 2% of the markers mapped to more than one locus in the genome. The high percentage (mostly above 70%) accumulation for axis 1 and axis 2 in all PCO and PCoA analyses of DArT markers in both case studies provides additional support that this expectation is justified. DNA sequencing of DArT markers confirmed that redundancy between different DArT markers is low. Some DArT markers, however, are still likely to be erroneously scored as independent if they originated from partially overlapping genomic regions. DArT marker DNA sequences suggest that very few DArT markers are mistakenly treated as independent and further comfort is derived from the observation that all three plant genomes contributed to the DArT data set.

A related problem is the assumption of homology when DArT markers are absent and data are analysed in a parsimony framework [35]. Similar to other presence/absence data (AFLP, ISSR), homology assessment is restricted to the hypothesis that the state of presence is the result of presence of homologous markers but the state of absence may be the result of alternative processes [35]. Currently, it is not possible to evaluate the putative misleading effects of such a bias. A further restriction of presence/absence data is the limited number of character states (two). However, this problem is likely to be offset by the large number of DArT markers available [35], which exceeds several hundred for both case studies presented here.

Distance-based methods are putatively less affected by these problems because some similarity scores take data structure and heritage (which in DArT is likely dominant) into account. Thus, Nei-Li similarity score [36] is probably the most appropriate for DArT data, for the same reasons given for AFLP data, i.e. presence/absence data structure and dominant inheritance [37]. This is an advantage of using the DArT technique because existing software tools designed for analyses of AAD markers can be easily adjusted to analyse DArT data. However, users should keep in mind the existence of modified versions of these distance measures [37]. The selection of tree building algorithms, for example UPGMA and Neighbor-Joining (NJ) is also important in the context of distance-based approaches. UPGMA is likely to result in incorrect topology as the result of the imbalanced distribution of variation among putative branches in the tree. Recently developed approaches such as NeighborNet and split decomposition analyses as implemented in Splitstree [28] are likely to be more powerful approaches for phylogenetic reconstruction based on DArT data transformed into appropriate distance matrices. Importantly, DArT procedures hold the potential to generate co-dominant markers by taking into account the strength of the signal for each DArT marker. This improvement could potentially overcome several restrictions limiting the analyses of AAD markers and current (dominant) DArT markers.

In general, character-based approaches are a more powerful tool to assess phylogenetic patterns and some authors therefore argue for their superiority [35]. Most parsimony analyses are now commonly used in addition to distance based approaches to evaluate AAD data sets, and these analyses should also be employed to analyse DArT data sets. More recently, Bayesian inference of phylogeny was made available by implementing a restriction site (binary) model based on an F81-like model into MrBayes 3.01 [26]. This model can also be used to analyse DArT marker data sets due to the underlying assumptions for the restriction sites model, non-observable absence data for example, fitting the structure of DArT data.

### Practical advantages of DArT in evolutionary research

The results reported here provoke the question: what are the practical advantages and disadvantages of using DArT (as opposed to other methods) to detect genomic variation for analysis in an evolutionary or phylogenetic framework? DArT offers seven key advantages over other widely used methods for detecting molecular variation: First, DArT is a sequence-independent discovery tool that requires no preliminary sequence information such as the identification of candidate loci or time-intensive development and optimization of primers. Thus DArT is likely to find variable sequences that other sequence-dependent technologies might miss. Second, DArT recovers a high level of variation, and offers the potential for very high throughput analysis of both markers and specimens. Third, the presence of a DArT marker is determined by DNA-DNA hybridization rather than fragment length and hence DArT suffers less from ambiguous homology assessments than other finger-printing methods utilising AAD markers, especially RAPDs, ISSRs and AFLPs [4]. Indeed, independent segregation of DArT markers in mapping experiments in barley [12] as well as in other species like wheat and sorghum (unpublished data) provides strong evidence for this point. Fourth, DArT is highly reproducible; DArT marker scoring is consistent among independent replicates (this study and [7]), indicating that DArT may be a more reliable genotyping method than other AAD markers [4]. Fifth, redundancy between DArT markers is both quantifiable and low (this study and [7]). Sixth, DArT markers are very easily sequenced (no gel isolation required), allowing similarity searches against sequence databases and internal redundancy estimates. Twenty-four percent of *Asplenium* and 19% of *Garovaglia* DArT marker DNA sequences yielded GenBank alignments including known mitochondrial, chloroplast and nuclear loci, proteins and predicted expressed genes (Table 4). Amongst these were matches to *rpl2, psbA* and *rps3* genes, and the locus encoding G3PDH, all of which are routinely used in sequence-based evolutionary studies in plants [38]–[44]. This demonstrates that DArT is capable of identifying known polymorphic loci, and suggests that the sequences of the hundreds of other unknown polymorphic fragments recovered by DArT may facilitate primer design for the amplification of novel target loci for direct sequencing. Finally, DArT is able to detect both genetic hybrids and chimeric environmental samples, demonstrating its utility for studies exploring the origin of hybrids and their specific genetic structure.

### Evolutionary insights derived from this study

Analyses of *Asplenium* and *Garovaglia* DArT data enabled the reconstruction of intraspecific structure in *A. viride* and *G. elegans* and revealed substrate specificity and/or phylogeographic patterns which, for *A. viride,* were not detectable using chloroplast or nuclear DNA sequences. All analyses of the *Asplenium* DArT data indicated a widespread limestone genotype group and a second limestone genotype group occurring in northern Africa and the Iberian Peninsula, incorporating two magnesit samples from Austria as putative relatives (Figures 1, 2, 3). The phylogeographic and substrate specificity patterns detected in analyses of *A. viride* DArT data (Figures 1 and 2) provoke at least two alternative scenarios of substrate evolution in *A. viride*: (1) *A. viride* originated on serpentine and twice colonized limestone habitats or (2) the extant serpentine populations of *A. viride* are relicts able to survive on a less favorable substrate. Both scenarios are consistent with the lack of alleles private to serpentine populations of *A. viride* (unpublished data). For *Garovaglia,* both DArT and cpDNA data clearly separated the Australian from the Papua New Guinean samples (Figure 4). When contrasted against the geographical distinction between Papua New Guinean and Australian *G. elegans* (ssp. *dietrichiae*, endemic to Australia) specimens, the lack of micro-biogeographic structure detected in Australian ssp. dietrichiae clade (Figures 1 and 2) suggests a high rate of gene exchange among the Australian populations (which may be a reflection of its nanandrous breeding system) or a low rate of gene exchange between Australian and New Guinean populations. Lastly, DArT enabled the detection of additive variation caused by genetic hybridization (*A. adulterinum*) or a mixed-taxon specimen (*G. elegans* Gel6434).

In conclusion, while DArT has been previously used to complement existing technologies in crop breeding and genomics [10], our studies show that the abundant non *a priori* comparative molecular data generated by DArT also holds real potential for use in a wide range of high-throughput evolutionary studies. These include the detection of biological correlations and phenomena including similarity, hybridization, mixed environmental samples, ecological differentiation and geographical distribution, as well as other studies of non-model organisms including gene discovery, QTL mapping, population and conservation genetics, speciation and environmental forensics.

## Supporting Information

Table S1 DArT markers, specimen genotypes and sequence-associated data. This supplementary table contains 1) detailed information about each of the 1349 DArT markers recovered for this study including marker-specific library preparation data and statistics, 2) DArT genotyptes of *Asplenium* and *Garovaglia* specimens, and 3) sequence-associated data from each of the 148 sequenced DArT markers including GenBank accession numbers, fragment lengths, blastn and tblastx identifications, % identity (closest match), and number different species hit for same function.
(0.50 MB XLS)

## References

[1] Avise JC (2004). Molecular markers, natural history, and evolution..

[2] Ranz JM, Machado CA (2006). Uncovering evolutionary patterns of gene expression using microarrays.. Trends in Ecology & Evolution.

[3] Gilad Y, Borevitz J (2006). Using DNA microarrays to study natural variation.. Current Opinion in Genetics & Development.

[4] Bussell JD, Waycott M, Chappill JA (2005). Arbitrarily amplified DNA markers as characters for phylogenetic inference.. Perspectives in Plant Ecology Evolution and Systematics.

[5] Kilian A, Huttner E, Wenzl P, Jaccoud D, Carling J, Tuberosa R, Phillips RL, Gale M (2003). The fast and the cheap: SNP and DArT-based whole genome profiling for crop improvement.. In the Wake of the Double Helix: From the Green Revolution to the Gene Revolution.

[6] Wittenberg AHJ, van der Lee T, Cayla C, Kilian A, Visser RGF (2005). Validation of the high-throughput marker technology DArT using the model plant Arabidopsis thaliana.. Molecular Genetics and Genomics.

[7] Wenzl P, Carling J, Kudrna D, Jaccoud D, Huttner E (2004). Diversity Arrays Technology (DArT) for whole-genome profiling of barley.. Proceedings of the National Academy of Sciences of the United States of America.

[8] Jaccoud D, Peng K, Feinstein D, Kilian A (2001). Diversity Arrays: a solid state technology for sequence information independent genotypinG.. Nucleic Acids Research.

[9] Lezar S, Myburg AA, Berger DK, Wingfield MJ, Wingfield BD (2004). Development and assessment of microarray-based DNA fingerprinting in Eucalyptus grandis.. Theoretical and Applied Genetics.

[10] Xia L, Peng KM, Yang SY, Wenzl P, de Vicente MC (2005). DArT for high-throughput genotyping of Cassava (Manihot esculenta) and its wild relatives.. Theoretical and Applied Genetics.

[11] Akbari M, Wenzl P, Caig V, Carling J, Xia L (2006). Diversity arrays technology (DArT) for high-throughput profiling of the hexaploid wheat genome.. Theoretical and Applied Genetics.

[12] Wenzl P, Li HB, Carling J, Zhou MX, Raman H (2006). A high-density consensus map of barley linking DArT markers to SSR, RFLP and STS loci and agricultural traits.. BMC Genomics.

[13] Yang SY, Pang W, Ash G, Harper J, Carling J (2006). Low level of genetic diversity in cultivated pigeonpea compared to its wild relatives is revealed by diversity arrays technology.. Theoretical and Applied Genetics.

[14] Lovis JD, Reichstein T (1967). Über das spontane Entstehen von *Asplenium adulterinum* aus einem natürlichen Bastard.. Die Natuerwissenschaften.

[15] Doyle J, Doyle J (1987). A rapid DNA isolation procedure for small quantities of fresh leaf tissue.. Phytochem. Bull..

[16] Trewick SA, Morgan-Richards M, Russell SJ, Henderson S, Rumsey FJ (2002). Polyploidy, phylogeography and Pleistocene refugia of the rockfern *Asplenium* ceterach: evidence from chloroplast DNA.. Molecular Ecology.

[17] Diatchenko L, Lau YFC, Campbell AP, Chenchik A, Moqadam F (1996). Suppression subtractive hybridization: A method for generating differentially regulated or tissue-specific cDNA probes and libraries.. Proceedings of the National Academy of Sciences of the United States of America.

[18] Anderson JA, Churchill GA, Autrique JE, Tanksley SD, Sorrells ME (1993). Optimizing Parental Selection for Genetic-Linkage Maps.. Genome.

[19] Taberlet P, Gielly L, Pautou G, Bouvet J (1991). Universal Primers for Amplification of 3 Noncoding Regions of Chloroplast DNA.. Plant Molecular Biology.

[20] Schneider H, Ranker T, Russell SJ, Cranfill R, Geiger JMO (2005). Origin of the endemic fern genus Diellia coincides with the renewal of Hawaiian terrestrial life in the Miocene.. Proceedings of the Royal Society B-Biological Sciences.

[21] Ishikawa H, Watano Y, Kano K, Ito M, Kurita S (2002). Development of primer sets for PCR amplification of the PgiC gene in ferns.. Journal of Plant Research.

[22] Pedersen N, Newton AE, Newton AE, Tangney R, DeLuna E (2007). Phylogenetic and morphological studies within the Ptychomniales, with emphasis on the evolution of dwarf males.. Pleurocarpous Mosses: Systematics and Evolution.

[23] Altschul SF, Madden TL, Schaffer AA, Zhang JH, Zhang Z (1997). Gapped BLAST and PSI-BLAST: a new generation of protein database search programs.. Nucleic Acids Research.

[24] Hammer O, Harper D, Ryan P (2001). PAST: Palaeontological Statistics Software package for education and data analysis.. Palaeontologia Electronica.

[25] Maddison WP, Maddison DR (1989). Interactive Analysis of Phylogeny and Character Evolution Using the Computer-Program Macclade.. Folia Primatologica.

[26] Huelsenbeck JP, Ronquist F (2001). MRBAYES: Bayesian inference of phylogenetic trees.. Bioinformatics.

[27] Swofford DL (1993). Paup-a Computer-Program for Phylogenetic Inference Using Maximum Parsimony.. Journal of General Physiology.

[28] Bryant D, Moulton V (2002). NeighborNet: An agglomerative method for the construction of planar phylogenetic networks. In: Algorithms in Bioniformatics: Second International Workshop, WABI 2002, Rome, Italy, September 17–21, 2002. Proceedings..

[29] Rambaut A, Drummond A (2004). TRACER, version 1.2..

[30] Vandepeer Y, Dewachter R (1993). Treecon-a Software Package for the Construction and Drawing of Evolutionary Trees.. Computer Applications in the Biosciences.

[31] Vogel JC (1995). Multiple origins of polyploids in European Asplenium (Pteridophyta)..

[32] Vogel JC, Russell SJ, Rumsey FJ, Barrett JA, Gibby M (1998). On hybrid formation in the rock fern Asplenium x alternifolium (Aspleniaceae, Pteridophyta).. Botanica Acta.

[33] Pelser PB, Gravendeel B, van der Meijden R (2003). Phylogeny reconstruction in the gap between too little and too much divergence: the closest relatives of Senecio jacobaea (Asteraceae) according to DNA sequences and AFLPs.. Molecular Phylogenetics and Evolution.

[34] Koopman WJM (2005). Phylogenetic signal in AFLP data sets.. Systematic Biology.

[35] Simmons MP, Zhang LB, Webb CT, Muller K (2007). A penalty of using anonymous dominant markers (AFLPs, ISSRs, and RAMS) for phylogenetic inference.. Molecular Phylogenetics and Evolution.

[36] Nei M, Li WH (1979). Mathematical Model for Studying Genetic Variation in Terms of Restriction Endonucleases.. PNAS.

[37] Archibald JK, Mort ME, Crawford DJ, Santos-Guerra A (2006). Evolutionary relationships within recently radiated taxa: comments on methodology and analysis of inter-simple sequence repeat data and other hypervariable, dominant markers.. Taxon.

[38] Laroche J, Bousquet J (1999). Evolution of the mitochondrial rps3 intron in perennial and annual angiosperms and homology to nad5 intron 1.. Molecular Biology and Evolution.

[39] Olsen KM (2004). SNPs, SSRs and inferences on cassava's origin.. Plant Molecular Biology.

[40] Stefanovic S, Olmstead RG (2004). Testing the phylogenetic position of a parasitic plant (Cuscuta, Convolvulaceae, Asteridae): Bayesian inference and the parametric bootstrap on data drawn from three genomes.. Systematic Biology.

[41] Howarth DG, Baum DA (2005). Genealogical evidence of homoploid hybrid speciation in an adaptive radiation of Scaevola (Goodeniaceae) in the Hawaiian Islands.. Evolution.

[42] Kress WJ, Wurdack KJ, Zimmer EA, Weigt LA, Janzen DH (2005). Use of DNA barcodes to identify flowering plants.. Proceedings of the National Academy of Sciences of the United States of America.

[43] Koehler-Santos P, Lorenz-Lemke AP, Muschner VC, Bonatto SL, Salzano FM (2006). Molecular genetic variation in Passiflora alata (Passifloraceae), an invasive species in southern Brazil.. Biological Journal of the Linnean Society.

[44] Dane F, Liu JR, Zhang CK (2007). Phylogeography of the bitter apple, Citrullus colocynthis.. Genetic Resources and Crop Evolution.

